# Clinical Efficacy of Composite Autologous Chylated Fat and Concentrated Growth Factors for Treating Postinjection Alopecia After Facial Fillers: A Single‐Center Retrospective Study

**DOI:** 10.1111/jocd.70269

**Published:** 2025-06-12

**Authors:** Shuo Tang, Guiwen Zhou, Qiang Fu, Minliang Chen

**Affiliations:** ^1^ Senior Department of Burns and Plastic Surgery The Fourth Medical Center of Chinese PLA General Hospital Beijing 中国 China; ^2^ Chinese PLA Medical School Beijing China

**Keywords:** alopecia, autologous fat, concentrated growth factors, dermal fillers, esthetic medicine, filler complications

## Abstract

**Background:**

Postinjection alopecia is a rare but significant complication of facial fillers, likely caused by compromised microcirculation and follicular damage. Effective treatments are needed, but regenerative therapies specifically for this etiology lack systematic investigation.

**Objective:**

This study evaluated the efficacy and safety of combined autologous chylated fat (ACF) and concentrated growth factors (CGF) for treating acute alopecia following facial filler injection.

**Methods:**

In this single‐center, retrospective case series, nine female patients with acute alopecia (2–4 weeks post‐HA filler) were treated between June 2021 and June 2024. Treatment involved three monthly subcutaneous injections of a 1:1 ACF and liquid CGF mixture (0.1 mL/cm^2^). Outcomes were assessed at 6 months posttreatment initiation, including hair density, diameter, terminal/vellus ratio, anagen/telogen ratio, Global Aesthetic Improvement Scale (GAIS), and adverse events.

**Results:**

All nine patients completed the 6‐month follow‐up. Significant improvements (*p* < 0.001) were observed at 6 months compared to baseline: hair density increased from 32.7 ± 13.4 to 92.4 ± 23.4 hairs/cm^2^, hair diameter from 52.3 ± 15.2 to 75.8 ± 21.7 μm, terminal/vellus ratio from 0.31 ± 0.11 to 1.27 ± 0.35, and anagen proportion from 18.4% ± 6.5% to 63.7% ± 14.9%. GAIS scores indicated high satisfaction. Treatment was well‐tolerated, with only one patient reporting transient erythema.

**Conclusion:**

Combined ACF and CGF represent a promising and effective treatment for post‐filler alopecia, offering a novel therapeutic option for early intervention and improved esthetic outcomes.

## 1 Introduction

Injectable fillers are increasingly utilized for nonsurgical facial rejuvenation. Although offering esthetic benefits, they carry risks, including iatrogenic alopecia in the temporal‐frontal region. Though rare, this complication is distressing due to potential permanent follicular damage, psychological impact, and medical disputes [1], underscoring the need for effective treatments.

Filler‐induced alopecia has a multifactorial etiology, primarily involving vascular compromise, inflammation, and mechanical compression. Accidental intra‐arterial injection can cause dermal papilla embolism and ischemia–reperfusion injury [2]. Filler materials may also incite delayed‐type hypersensitivity, leading to chronic granulomatous inflammation and cicatricial alopeia [3]. Additionally, edema or excessive filler volume can compress hair follicles, disrupting their cycling, particularly in high‐tension scalp areas like the frontal hairline [4].

Current management for post‐filler alopecia includes hyaluronidase, intralesional corticosteroids, topical minoxidil, platelet‐rich plasma (PRP), and hyperbaric oxygen therapy [5, 6]. However, these options show variable efficacy and have limitations. For instance, hyaluronidase efficacy diminishes rapidly postvascular event, and prolonged corticosteroid use carries adverse risks.

Bioactive materials represent a promising avenue for alopecia treatment. Platelet‐concentrated products (e.g., PRP, CGF) promote perifollicular angiogenesis via growth factors like VEGF and PDGF‐BB [7]. Adipose‐derived components, like stromal vascular fraction (SVF) and extracellular matrix (ECM), enhance the follicular microenvironment through paracrine signaling from adipose‐derived stem cells (ADSCs), activating pathways such as Wnt/β‐catenin and ERK to shift follicles from telogen to anagen [8, 9]. Notably, Butt et al. [10] demonstrated a 51.64% improvement in hair density in androgenetic alopecia with combined PRP and SVF, surpassing monotherapy and supporting combination strategies.

Despite these advances, limitations in managing post‐filler alopecia persist, including a lack of tailored acute‐phase protocols, challenges with traditional adipose transplantation (e.g., poor volume retention, low graft survival), and rapid dissipation of bioactive components from platelet concentrates. To address these limitations, we developed a novel composite gel combining ACF and CGF, leveraging physical emulsification and biological cross‐linking for stabilized follicular microenvironment regulation.

## 2 Materials and Methods

### 2.1 Study Design and Ethical Oversight

This single‐center, retrospective case series was conducted in accordance with the Strengthening the Reporting of Observational Studies in Epidemiology (STROBE) guidelines. The study protocol was reviewed and approved by the Institutional Review Board (IRB) of the Fourth Medical Center of the People's Liberation Army General Hospital (approval no.: 2024YL039‐KS001). Written informed consent was obtained from all participants prior to enrollment, after a comprehensive explanation of the study's objectives, methodology, and potential risks. The study adhered to the ethical principles outlined in the Declaration of Helsinki.

### 2.2 Participant Selection

Inclusion Criteria: Participants were eligible if they were ≥ 18 years of age; experienced temporal or frontal alopecia onset within 2–4 weeks following hyaluronic acid filler injection; exhibited dermatoscopic evidence of ischemic changes, including perifollicular erythema, hair shaft fractures, and the white dot sign; and had no prior history of androgenetic alopecia, alopecia areata, or other forms of cicatricial alopecia. Exclusion criteria: Individuals with coagulation disorders or a platelet count < 100 × 10^9^/L; a history of alopecia‐related treatments (e.g., minoxidil, platelet‐rich plasma therapy) within the preceding 6 months; or an active infection at the injection site were excluded.

### 2.3 Preparation and Injection of ACF–CGF Complex

**FIGURE 1:**
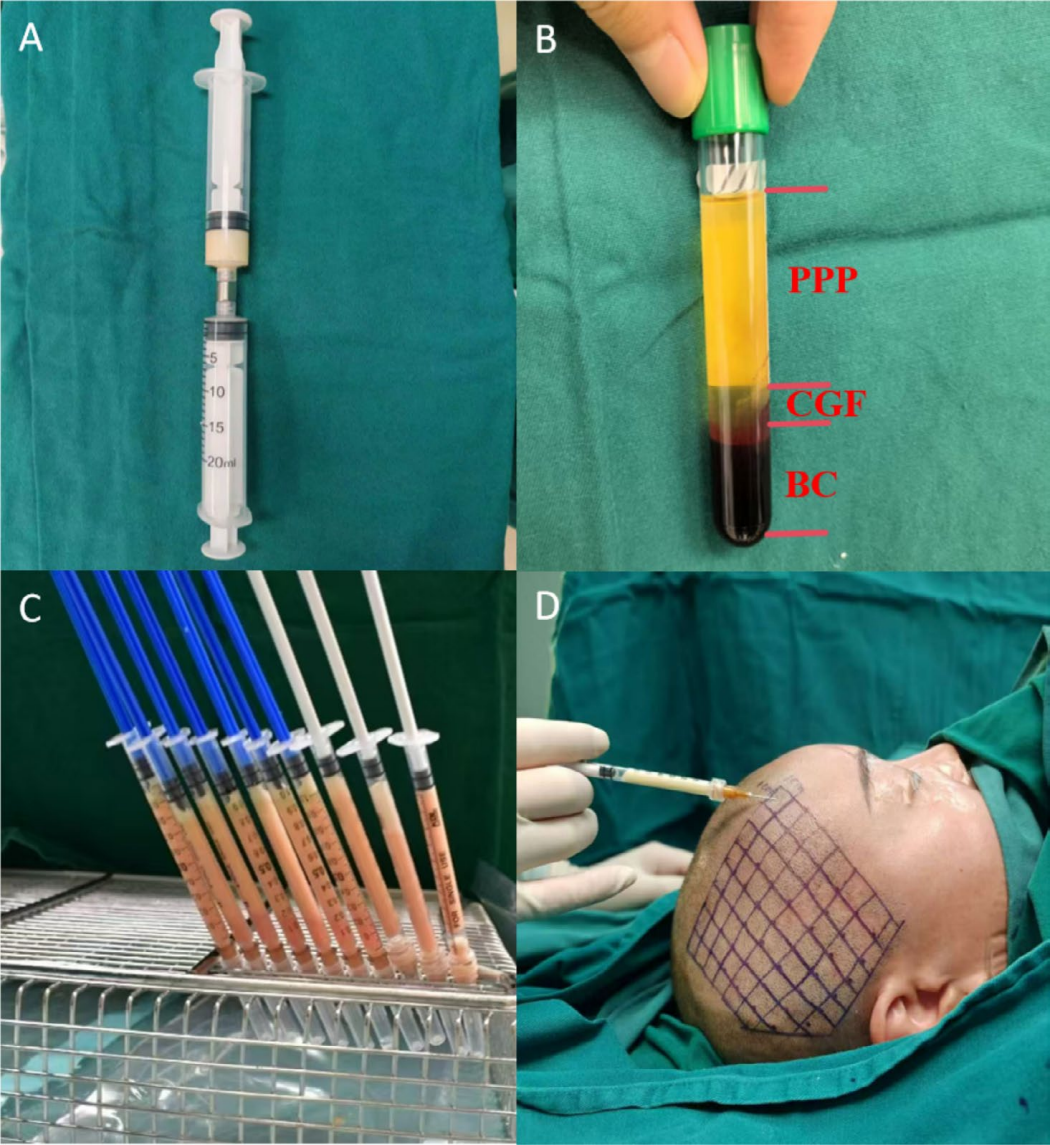
A 35‐year‐old female patient with temporal alopecia (case 1) (A) before treatment and (B) 6 months after last treatment. (A) Chylated fat generated using a 0.8 mm processing adapter; (B) Whole blood after centrifugation showing three distinct fractions. Bottom layer: dark mixture of erythrocytes and leukocytes called buffy coat (BC). Middle layer: thin, translucent fraction enriched with concentrated growth factors (CGF). Top layer: yellowish platelet‐poor plasma (PPP). (C) 1:1 mixture of ACF and CGF. (D) Mark the alopecia area with a 1‐cm^2^ grid. Inject the ACF–CGF premixture subcutaneously into each grid square at 0.1 mL/cm^2^.

Autologous adipose tissue was harvested from the lower abdomen using the Coleman technique at the beginning of each of the three treatment sessions [11]. The harvested tissue underwent sequential filtration, washing, and purification. Mechanical emulsification was performed by repeatedly passing the tissue through a 0.8 mm nanopore device, resulting in homogenized adipose fragments with particle sizes < 0.5 mm (Figure 1A). Venous blood (10 mL) was collected in VACUETTE CGF tubes (Greiner Bio‐One, Austria) and centrifuged using a Medifuge 2000 system (Italy) according to the following protocol: 2700 rpm for 2 min, 2400 rpm for 4 min, 2700 rpm for 4 min, and 3000 rpm for 3 min. The resulting liquid CGF layer was isolated (Figure 1B). The ACF and liquid CGF were combined in a 1:1 volume ratio and allowed to rest for 5 min to facilitate fibrin cross‐linking, forming a composite gel (Figure 1C). This gel was injected subcutaneously into the alopecia‐affected area using a 23G blunt cannula, at a depth of 1–2 mm. The treatment area was divided into grid‐based subunits, with each subunit receiving 0.1 mL/cm^2^ of the gel (Figure 1D). Postinjection, a cold compress was applied for 10 min. Participants received monthly injections for three consecutive months, with a 6‐month follow‐up period after the final treatment.

### 2.4 Evaluation Criteria

Clinical assessments were performed at baseline (pretreatment), 3 months posttreatment, and 6 months posttreatment. Hair density was calculated by manually counting hairs within a defined 1 cm^2^ area, using a standardized photographic system. This process was repeated three times, and the results were averaged. The mid‐shaft diameter of 10 representative hairs was measured using a digital microscope. Follicle function was assessed by determining the ratio of anagen (growing) to telogen (resting) hairs. Anagen hairs were defined as having a shaft length ≥ 3 mm and root pigmentation. The ratio of terminal‐to‐vellus hairs was measured to evaluate follicular structure. Terminal hairs were defined as having a diameter ≥ 40 μm, a shaft length ≥ 3 mm, and root pigmentation. Esthetic outcomes were evaluated using the GAIS (Table 1). Two independent physicians compared posttreatment photographs to baseline images. Patient satisfaction was assessed using a self‐assessment survey. Adverse events (e.g., redness, swelling, infection) were recorded and graded using the Clavien‐Dindo classification system.

**TABLE 1:** The Global Aesthetic Improvement Scale (GAIS) of the efficacy evaluation for five degrees.

Score	State	Description
3	Very much improved	Optimal esthetic result for the treatment in this patient.
2	Much improved	Marked improvement in hair appearance, but not completely optimal, and a touch‐up would improve the result.
1	Improved	Improvement in hair appearance from the original condition, but a touch‐up is advised.
0	No change	The hair appearance is essentially the same compared with the original condition.
−1	Worse	The hair appearance is worse than the original condition.

### 2.5 Statistical Analysis

Data were analyzed using SPSS software (version 24.0, IBM Corp.). Descriptive statistics are presented as mean ± standard deviation (SD). A one‐way repeated measures ANOVA was used to analyze hair density, shaft diameter, terminal‐to‐vellus hair ratio, and anagen hair ratio. Bonferroni‐corrected post hoc tests were performed for pairwise comparisons. The Wilcoxon signed‐rank test was used to assess GAIS scores. Statistical significance was defined as *p* < 0.05, and *p* < 0.01 was considered highly significant.

## 3 Result

### 3.1 Baseline Characteristics and Treatment Adherence

Nine female patients (mean age: 33.7 ± 6.6 years; range: 24–45 years) presenting with acute alopecia in either the temporal (*n* = 5) or frontal (*n* = 4) region following hyaluronic acid injection were enrolled. The mean duration of alopecia prior to intervention was 22.8 ± 5 days (Table 2). All patients completed the prescribed treatment regimen, consisting of three sessions of ACF–CGF composite gel injections, and were followed for 6 months. No patient attrition occurred during the study period.

**TABLE 2:** Characteristics of the study patients.

No	Sex/age (years)	Hair loss duration (days)	Affected area	Previous treatments
1	F/28	18	Temporal	None
2	F/35	25	Temporal	None
3	F/40	30	Temporal	None
4	F/24	15	Temporal	None
5	F/31	20	Temporal	None
6	F/45	28	Frontal	None
7	F/29	19	Frontal	None
8	F/33	24	Frontal	None
9	F/38	26	Frontal	None

Quantitative and qualitative assessment of therapeutic efficacy follow‐up data demonstrated statistically significant improvements in hair density at both 3 and 6 months posttreatment. Hair density increased from a baseline of 32.7 ± 13.4 hairs/cm^2^ to 48.2 ± 18.2 hairs/cm^2^ at 3 months (*p* = 0.002) and 92.4 ± 23.4 hairs/cm^2^ at 6 months (*p* < 0.001). A corresponding significant increase in hair shaft diameter was observed, rising from 52.3 ± 15.2 μm at baseline to 64.1 ± 19.8 μm (*p* = 0.003) and 75.8 ± 21.7 μm (*p* < 0.001) at 3 and 6 months, respectively, suggestive of hair shaft remodeling. Dynamic trichoscopic analysis revealed a significant increase in the proportion of anagen hairs, from 18.4% ± 6.5% at baseline to 45.2% ± 11.4% (*p* < 0.001) and 63.7% ± 14.9% (*p* = 0.002) at the respective follow‐up time points. Furthermore, clinical signs of follicular ischemia, including white dot sign and perifollicular erythema, were completely resolved. The terminal‐to‐vellus hair ratio improved significantly from 0.31 ± 0.11 at baseline to 0.75 ± 0.23 (*p* = 0.015) and 1.27 ± 0.35 (*p* < 0.001). Physician and patient‐reported outcomes demonstrated significantly greater therapeutic effects at 6 months compared to 3 months (*p* < 0.05), indicating progressive and sustained improvement over the treatment duration (Table 3). The treatment was well‐tolerated. One patient (11.1%) experienced transient, localized erythema (Clavien‐Dindo Grade I) following the initial injection, which resolved within 24 h with conservative management (cold compresses). No serious adverse events, including nodule formation, infection, or vascular compromise, were observed during the follow‐up period.

**TABLE 3:** Relevant change of hair growth parameters, GAIS of physicians and patients.

	Baseline	3 Months	6 Months	*p*	*p* ^a^	*p* ^b^
**Hair parameter**						
Hair density (hairs/cm^2^)	32.7 ± 13.4	48.2 ± 18.2	92.4 ± 23.4	0.002	< 0.001	0.001
Hair diameter (μm)	52.3 ± 15.2	64.1 ± 19.8	75.8 ± 21.7	0.003	< 0.001	0.012
Anagen hair ratio (%)	18.4 ± 6.5	45.2 ± 11.4	63.7 ± 14.9	< 0.001	0.002	0.002
T/V ratio	0.31 ± 0.11	0.75 ± 0.23	1.27 ± 0.35	0.015	< 0.001	0.008
**GAIS (physicians)**						
−1 (*n*/%)	—	0 (0)	0 (0)			
0 (*n*/%)	—	2 (0.22)	0 (0)			
1 (*n*/%)	—	6 (0.66)	5 (0.55)			
2 (*n*/%)	—	1 (0.11)	3 (0.33)			
3 (*n*/%)	—	0 (0)	1 (0.11)			
Average GAIS	—	0.89 ± 0.57	1.56 ± 0.69			0.016
**GAIS (patients)**						
−1 (*n*/%)	—	0 (0)	0 (0)			
0 (*n*/%)	—	1 (0.11)	0 (0)			
1 (*n*/%)	—	7 (0.77)	4 (0.44)			
2 (*n*/%)	—	1 (0.11)	3 (0.33)			
3 (*n*/%)	—	0 (0)	2 (0.22)			
Average GAIS	—	1.00 ± 0.47	1.78 ± 0.79			0.034

*Note:* *p* denotes comparisons between 3 months and baseline.

^a^ *p* denotes comparisons between 6 months and baseline.

^b^ *p* denotes comparisons between 6 months and 3 months.

### 3.2 Illustrative Case Presentations

#### 3.2.1 Case 1

**FIGURE 2:**
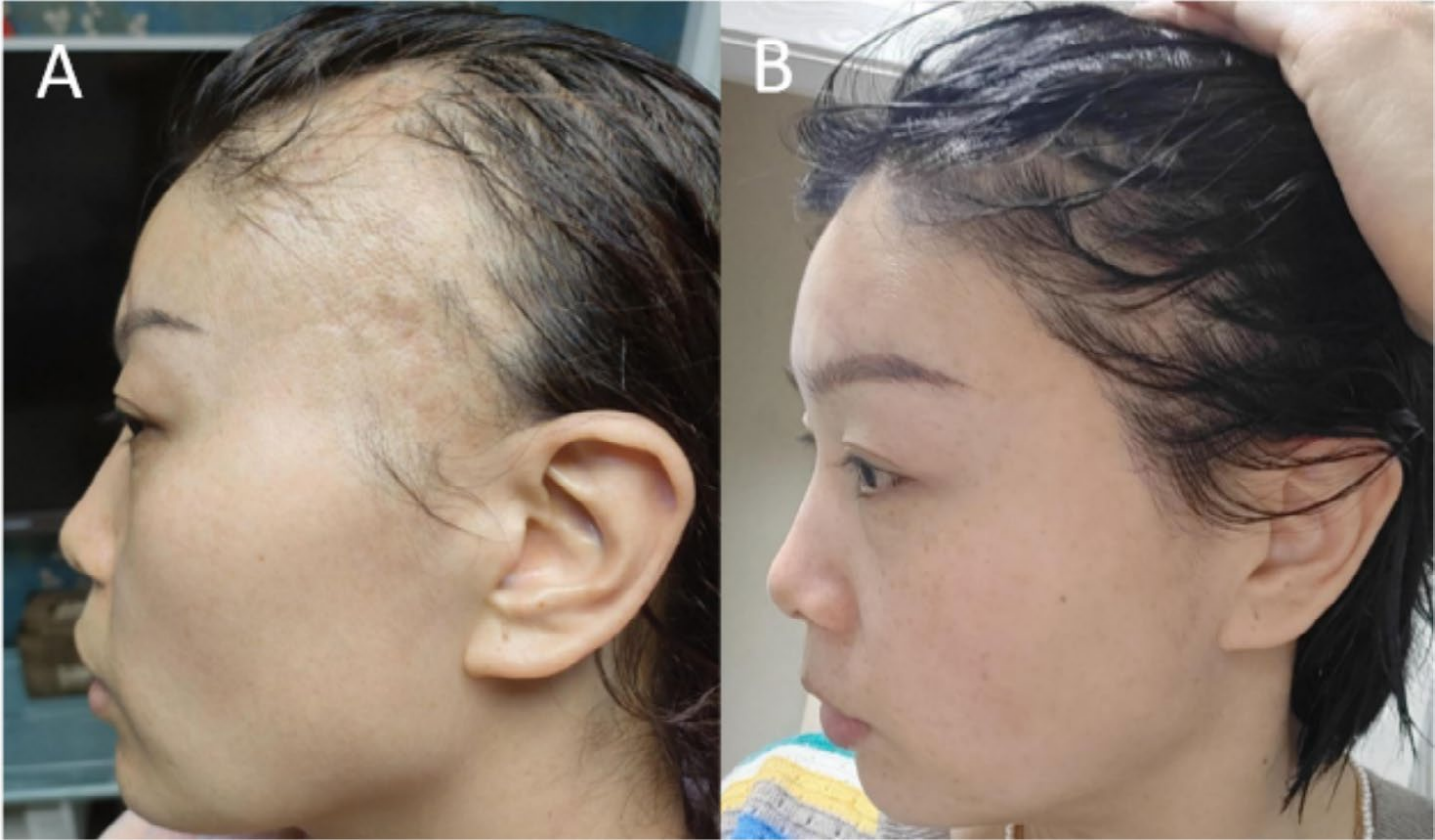
A 35‐year‐old female patient with temporal alopecia (case 1) (A) before treatment and (B) 6 months after last treatment.

A 35‐year‐old female presented with temporal alopecia 25 days post‐hyaluronic acid injection. Serial photographs (Figure 2A,B) demonstrate progressive hair regrowth at 6 months posttreatment.

#### 3.2.2 Case 2

**FIGURE 3:**
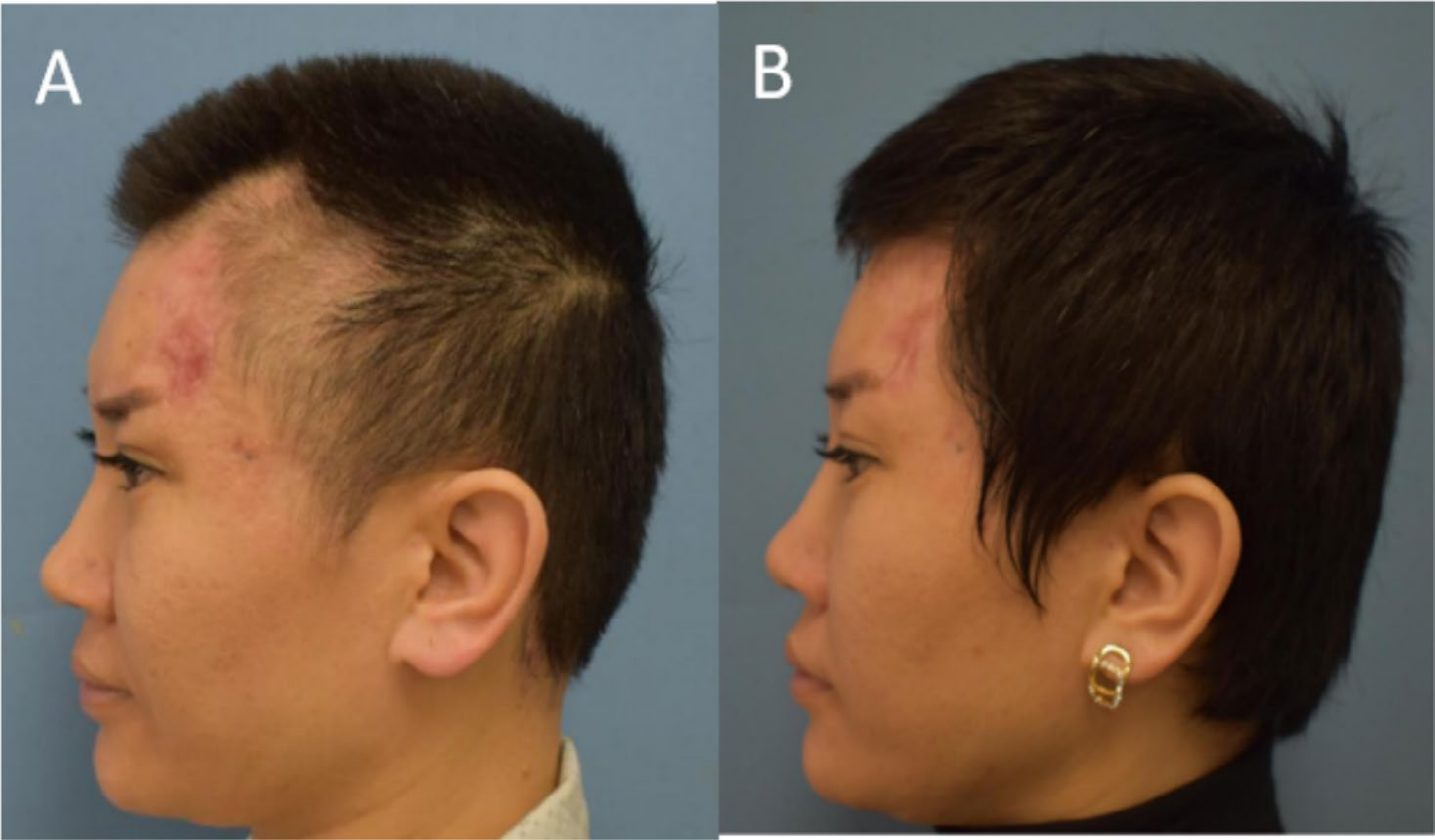
A 29‐year‐old female patient with temporal alopecia (case 2) (A) before treatment and (B) 6 months after last treatment.

A 29‐year‐old female presented with temporal alopecia 19 days post‐hyaluronic acid injection. Photographic documentation (Figure 3A,B) illustrates the hair regeneration process over the 6‐month treatment course.

## 4 Discussion

This study demonstrates the therapeutic efficacy of a novel composite gel, combining autologous conditioned fibrin (ACF) and concentrated growth factors (CGF), in the management of acute alopecia secondary to injectable fillers. At six‐month follow‐up, a statistically significant 183% increase in hair density was observed (from 32.7 ± 13.4 to 92.4 ± 23.4 hairs/cm^2^, *p* < 0.001). Concurrently, hair shaft diameter increased by 44.9% (from 52.3 ± 15.2 to 75.8 ± 21.7 μm, *p* < 0.001), and the terminal‐to‐vellus hair ratio demonstrated a 309.7% increase (from 0.31 ± 0.11 to 1.27 ± 0.35, *p* < 0.001). These quantitative improvements indicate substantial recovery from filler‐induced ischemic follicular injury.

The pathophysiology of postinjection alopecia involves a complex interplay of vascular compromise (thrombosis and mechanical compression), inflammatory dysregulation, and subsequent follicular ischemia and hypoxia [4]. In our study, all nine patients showed acute‐phase ischemic injury to the hair follicles (22.8 ± 5 days). This aligns with the thrombosis characteristics of the papillary vascular network caused by the misinjection of fillers into the superficial temporal artery, as reported by Xie et al. [5] Although early intervention with hyaluronidase is crucial for dissolving hyaluronic acid‐based fillers and mitigating vascular occlusion, it does not fully address the established ischemia–reperfusion injury or the disruption of the hair follicle stem cell niche. The ACF–CGF composite gel, however, directly addresses these multifaceted pathological processes by providing a regenerative microenvironment.

Our previous work has demonstrated the efficacy of ACF in treating hypertrophic scars [12] and CGF in enhancing wound healing and esthetic outcomes [13]. This study builds upon that foundation, applying this innovative combination to the specific challenge of filler‐induced alopecia. The specialized processing of ACF produces microparticles smaller than 0.5 mm in diameter. According to previous research [14], these particles form a three‐dimensional scaffold after subcutaneous injection; this scaffold likely helps stabilize the perifollicular extracellular matrix and reduces mechanical pressure from the expansion of fillers. Adipose transplantation thickens the subcutaneous fat layer, offering structural support and metabolic substrates to the hair follicles. Adipocytes surrounding the follicles are known to release hormones, such as leptin and adiponectin, which directly regulate follicular stem cell activity [15]. Furthermore, the differentiation of pre‐adipocytes into mature adipocytes can lead to BMP2 secretion, inhibiting follicular regression [16]. The ACF is rich in ADSCs and growth factors like VEGF and FGF, which foster angiogenesis, activate follicles, and promote tissue regeneration through paracrine mechanisms [17]. This ability to promote angiogenesis supplies essential nutrients to the follicles, improves the ischemic microenvironment, and can reverse follicular regression caused by vascular injury. ADSCs release various bioactive factors, such as IGF‐1, PDGF, EGF, and TGF‐β, which stimulate the proliferation and differentiation of follicular stem cells. For instance, IGF‐1 activates the anagen phase and extends the follicular cycle [18]. Research on androgenetic alopecia has shown that ADSC‐exosomes can promote DPC proliferation and increase the expression of cyclins, β‐catenin, versican, and BMP2. They also modulate the TGF‐β1/SMAD3 signaling pathway via miR‐122‐5p and counteract the inhibitory effects of DHT on hair growth [19, 20]. The inflammatory microenvironment in postinjection alopecia is characterized by elevated levels of proinflammatory cytokines, contributing to follicular damage. ADSCs within the ACF component possess immunomodulatory properties, suppressing proinflammatory factors (e.g., TNF‐α, IL‐6) and promoting anti‐inflammatory factors (e.g., IL‐10) [21]. Mesenchymal stem cells (MSCs) within the stromal vascular fraction (SVF) can further modulate T cell function, potentially mitigating autoimmune responses that can exacerbate hair loss [22]. The ACF–CGF combined therapy uses the anti‐inflammatory and regenerative properties of adipose stem cells along with growth factors from CGF, creating a dual mechanism that is particularly effective for treating inflammatory alopecia caused by fillers. These findings highlight the complex role of ACF in regulating the follicular microenvironment, leading to significant improvements in hair density and diameter after 6 months of treatment.

CGF, a third‐generation platelet concentrate rich in platelets, fibrin, and CD34^+^ stem cells, slowly releases growth factors like PDGF, VEGF, and TGF‐β through its fibrin network, creating a favorable environment for hair growth that lasts several weeks [23]. In our study, we administered injections once a month, which aligns with this release pattern. CD34^+^ stem cells in CGF may directly contribute to follicle repair and regeneration. They have the potential to differentiate and stimulate the proliferation of follicular epithelial cells, thus promoting the follicle's transition from the telogen phase back into the anagen phase [24]. Dynamic trichoscopy showed a notable increase in the percentage of anagen hairs, consistent with the effects of CGF on the follicle growth cycle. The growth factors released by CGF, such as PDGF and VEGF, promote the formation of new blood vessels, improve local blood circulation, and provide additional nutrition to the follicles [25]. Clinical trials [26, 27, 28, 29] for androgenetic alopecia have shown that CGF can significantly increase terminal hair density, follicular density, and hair diameter. These effects last for more than 6 months after treatment, whether CGF is used alone or in combination with other therapies. In a randomized controlled clinical study, Behrangi et al. [30] found that a single SVF treatment combined with two PRP injections had no significant difference in effectiveness compared to three PRP injections alone for treating AGA. The findings of Butt et al. [10] indicate that multiple interventions may be necessary for cell therapy to provide its full benefits. In this study, the effects of the ACF combined with CGF therapy clearly supported this conjecture. It is crucial to understand that the pathological mechanisms of hair loss after injection surgery mainly involve local disturbances in microcirculation and inflammatory responses, which differ from the hormone‐dependent nature of androgen alopecia. Further research is essential to better understand the specific mechanisms of action associated with these treatments.

This study has several limitations that warrant careful consideration.

Firstly, a key limitation is the lack of standardized, objective evaluation methods for quantifying hair regrowth. While manual hair counting and dermoscopy provided clinically meaningful data, future studies would benefit from incorporating automated trichoscopy (e.g., Folliscope) and molecular biomarkers (e.g., VEGF levels) for enhanced objectivity. Secondly, the small sample size (*n* = 9) limits the generalizability of the findings, and a larger, multi‐center randomized controlled trial (RCT) is needed to confirm these results with greater statistical power. Thirdly, the six‐month follow‐up period, while demonstrating significant short‐term improvements, does not allow for assessment of long‐term efficacy and safety, including hair maintenance rates and potential delayed complications. Lastly, the mechanistic insights, while supported by existing literature, are largely inferential and lack direct molecular validation within this study.

Future research should focus on: (1) employing single‐cell RNA sequencing to analyze the regulatory mechanisms of ACF–CGF on hair follicle stem cell subpopulations and the surrounding microenvironment; (2) creating animal models to simulate postinjection alopecia and assess the composite's effects on angiogenesis and inflammation; (3) optimizing the ACF–CGF ratio with concentration gradient experiments and exploring combinations with other biomaterials, including exosomes, to enhance therapy; and (4) incorporating rigorous objective outcome measures and extending follow‐up periods in future clinical investigations to comprehensively evaluate long‐term efficacy and safety.

## 5 Conclusion

This study introduces a novel composite gel system that combines autologous chylous fat (ACF) with concentrated growth factors (CGF), showing its effectiveness in reversing acute alopecia following filler injections. Mechanistically, the therapeutic effects may stem from ACF‐derived 3D fibrous scaffold support, sustained CGF growth factor release, and adipose stem cell paracrine modulation of follicular microenvironments. The protocol exhibited favorable safety and marked esthetic enhancement, offering a novel strategy for early‐stage iatrogenic alopecia intervention. To confirm the long‐term effectiveness and broader applicability of this treatment, further validation through multicenter randomized controlled trials (RCTs) and research is crucial to fully elucidate the underlying mechanisms and optimize this innovative therapy.

## Author Contributions

Shuo Tang and Guiwen Zhou (co‐first authors) contributed equally to the study's conceptualization and design, data acquisition and analysis, and initial manuscript preparation. Qiang Fu assisted with data collection and critically reviewed the manuscript. Minliang Chen (corresponding author) was responsible for the study's conception, overall supervision, project administration, and critical revision of the manuscript. All authors have read and approved the final version of the manuscript.

## Ethics Statement

The study protocol was reviewed and approved by the Institutional Review Board (IRB) of the Fourth Medical Center of the People's Liberation Army General Hospital (approval no.: 2024YL039‐KS001). Written informed consent was obtained from all participants prior to enrollment, after a comprehensive explanation of the study's objectives, methodology, and potential risks. The study adhered to the ethical principles outlined in the Declaration of Helsinki.

## Conflicts of Interest

The authors declare no conflicts of interest.

## Data Availability

The data that support the findings of this study are available from the corresponding author upon reasonable request.
